# Caries lesion remineralization with fluoride toothpastes and chlorhexidine - effects of application timing and toothpaste surfactant

**DOI:** 10.1590/1678-7757-2017-0499

**Published:** 2018-05-22

**Authors:** Sami A. Almohefer, John A. Levon, Richard L. Gregory, George J. Eckert, Frank Lippert

**Affiliations:** 1 Indiana University School of Dentistry, Department of Prosthodontics, Indianapolis, Indiana, USA.; 2 Hail University College of Dentistry, Department of Prosthodontics, Hail, Saudi Arabia.; 3 Indiana University School of Dentistry, Department of Biomedical and Applied Sciences, Indianapolis, Indiana, USA.; 4 Indiana University School of Medicine, Department of Biostatistics, Indianapolis, Indiana, USA.; 5 Indiana University School of Dentistry, Department of Cariology, Operative Dentistry and Dental Public Health, Indianapolis, Indiana, USA.

**Keywords:** Dental caries, Sodium dodecyl sulfate, Tooth remineralization, Toothpastes

## Abstract

Habitual toothbrushing with fluoridated toothpaste followed by rinsing with antibacterial mouthwashes is a method to maintain good oral hygiene and to diminish the occurrence and severity of dental caries and periodontal disease. However, our understanding of how antimicrobial agents in mouthwashes affect fluoride-mediated caries lesion remineralization is still poor.

**Objective:**

The objectives of this *in vitro* study were a) to determine the effects of the waiting period of chlorhexidine (CHX) rinsing after fluoride toothpaste use and b) to further determine the effect of the type of toothpaste surfactant [sodium dodecyl sulfate (SDS) or cocamidopropyl betaine (CAPB)] on caries lesion remineralization associated with CHX rinsing.

**Material and Methods:**

Caries lesions were formed in bovine enamel specimens and assigned to 10 treatment groups (n=18) based on Vickers surface microhardness (VHN). Lesions were then pH-cycled for 10 days with daily regimen comprised of twice daily toothpaste slurry treatments (1150 ppm fluoride, with SDS or CAPB), followed by CHX solution treatments [0, 15, 30 or 60 minutes following slurry treatment or no CHX treatment (negative control)]. VHN was measured again and the extent of lesion remineralization calculated (∆VHN).

**Results:**

∆VHN with SDS-toothpaste was significantly lower than with CAPB-toothpaste, indicating more remineralization for the CAPB-toothpaste. ∆VHN with 0-minute waiting time was significantly lower than with 30-minute waiting time and with negative control.

**Conclusions:**

The absence of CHX as an adjunct to fluoride toothpastes led to greater remineralization of enamel lesions compared with the immediate use of CHX treatment for both SDS- and CAPB-toothpastes. CAPB-toothpastes indicated significantly greater remineralization than SDS-toothpastes, and can be suggested for patients at high risk of caries. A 30-minute waiting time for CHX treatment is recommended after brushing.

## Introduction

Fluoride has long been recognized as it promotes caries lesion remineralization and inhibits demineralization of tooth surfaces subjected to acids related to the caries process^40^. Several systematic reviews have concluded that fluoride toothpastes prevent caries^24^ ^,^ ^35^. A Cochrane review of 79 caries clinical trials demonstrated a dose-response effect of fluoride toothpaste, with caries decreases of 23% for fluoride concentrations between 1,000 and 1,250 ppm and reductions of 36% for fluoride concentrations between 2,400 and 2,800 ppm^38^. Furthermore, Marinho, et al.^24^ (2003) found a 14% improvement in caries prevention for brushing twice *vs.* once daily.

Several caries clinical trials have demonstrated that oral care habits have a significant impact on fluoride efficacy; in addition to brushing frequency, post-brushing rinsing behavior was shown to diminish the anticaries benefits of toothbrushing with fluoride toothpaste^11^ ^,^ ^12^ ^,^ ^23^ ^,^ ^28^. When investigating the effect of rinsing behavior on post-brushing salivary fluoride levels, the highest fluoride concentrations were found when subjects did not expectorate the toothpaste slurry after brushing, but used it as a mouth rinse. On the other hand, rinsing with tap water, expectorating and swallowing the slurry resulted in reduced fluoride retention. Subsequent studies highlighted rinsing with water after toothbrushing has detrimental effects on intraoral fluoride levels^5^ ^,^ ^16^ ^,^ ^18^ ^,^ ^31^, which explains observations from abovementioned clinical trials.

Surfactants are one of the key ingredients in toothpastes. Surfactants are responsible for the foaming action and intraoral dispersion of toothpastes, as well as for the micellization of water-insoluble ingredients, such as flavors and organic anti-plaque/antigingivitis compounds. SDS, an anionic surfactant, is by far the most used surfactant in toothpastes. These surfactants are favored in toothpaste formulations due to their compatibility with other excipients and good foaming characteristics. However, SLS in particular has been, albeit only anecdotally, associated with canker sores^20^. This has led manufacturers to utilize other less irritating surfactants, such as sarcosinates (anionic) and cocamidopropyl betaine (CAPB; amphoteric). CAPB is less irritating than SLS, although at the expense of foaming ability^13^ ^,^ ^20^. Little research has been conducted on how surfactants affect fluoride delivery to the dental hard tissues and, ultimately, lesion remineralization. As surfactants can modulate the surface charge of hydroxyapatite and block binding sites for fluoride, surfactants in toothpastes may affect remineralization. A recent study^2^ provided some evidence on this matter, although further research is necessary.

Oral care regimens are not limited to toothbrushing with fluoride toothpaste only and can include a wide range of additional measures, such as flossing and use of a mouthwash. However, these practices and the use of mouthwash, in particular, vary considerably between individuals^,^ ^9^ ^,^ ^17^ ^,^ ^41^. Furthermore, mouthwashes can be divided depending on their purpose. Some contain agents for caries prevention, breath freshening, tartar prevention, enhanced stain removal and/or improved antimicrobial action.

Chlorhexidine (CHX) is often seen as the “gold standard” due to its antimicrobial action against a wide variety of organisms that has been shown to reduce the incidence of plaque-induced gingivitis^4^ ^,^ ^19^ ^,^ ^22^. CHX has a comprehensive spectrum of activity, including some lipophilic viruses, yeasts, gram-positive and -negative bacteria, and dermatophytes^14^. A typical oral care regimen for patients at high risk of caries and periodontal disease (which often occur together^25^) consists of the use of fluoride toothpaste (caries prevention) followed by rinsing with an antimicrobial mouthwash (prevention of periodontal disease). However, some studies have shown toothpaste excipients, such as SDS, can lower the antimicrobial effect of cationic antimicrobials, such as CHX or cetylpyridinium chloride^8^ ^,^ ^30^. Another study^8^ demonstrated that the waiting time between SDS and CHX applications is of great importance as the anti-plaque effect of CHX was significantly reduced when the time between SDS and CHX exposures was 30 minutes or less. A waiting time of at least two hours was required to not interfere with CHX activity^8^. CHX varnish has been shown to reduce *S. mutans* counts in both saliva and dental plaque for periods ranging from 4 to 89 weeks^29^. CHX is substantive on the tooth surface and was found to form a coating several micrometers thick^32^. However, there is little evidence of substantial diffusion of CHX into the enamel, either from the surface or via the enamel lamellae^32^. CHX also interacts with fluoride because of electrostatic attraction^26^.

CHX was investigated as an anticaries agent in the past, although results were inconclusive. Baca, et al.^6^ ^,^ ^7^ (2002,2004) found a varnish containing both CHX and thymol (1% each) was able to reduce dental caries in deciduous and permanent molars. Additionally, Du, et al.^15^ (2006) found the application of a 40% CHX varnish twice a year reduced dental caries in primary molars. Symington, et al.^33^ (2014) concluded that 10% CHX was highly effective in reducing caries in high-risk adults. However, O’Keefe^27^ (2012) did not show any caries reduction in an adult population using 10% CHX varnish. A recent systematic review on the role of CHX varnish or gel for caries prevention found little evidence to support or disprove its use, and no trials have been conducted using 0.12 - 0.2% CHX mouthwashes^37^.

Therefore, the aims of this *in vitro* study were to determine the effects of the waiting period of 0.12% CHX rinsing after fluoride toothpaste use and type of surfactant (SDS or CAPB) on caries lesion remineralization. The null hypotheses were that a) increasing the waiting time between fluoride toothpaste and CHX treatments; and b) the type of surfactant will have no effect on the ability of fluoride to remineralize early enamel caries lesions.

## Materials and methods

### Study design

Demineralized bovine enamel specimens with predetermined surface microhardness (VHN) were submitted to a 10-day pH-cycling model. During the pH-cycling phase, specimens were exposed to fluoride toothpaste slurries; one containing SDS, the other an amphoteric surfactant (CAPB), followed by a 0.12% CHX rinse at different time intervals after fluoride exposure with no CHX or any other rinse as a negative control. After completion of this phase, the extent of remineralization was determined using VHN.

### Enamel specimens

Enamel specimens were prepared as described previously^21^: bovine incisor teeth were dissected into 5×5 mm specimens from the buccal surfaces only by means of a Buehler Isomet low speed saw (Isomet, Buehler Ltd, Lake Bluff, IL, USA). The teeth were stored in deionized water saturated with thymol (0.1% w/v) during the sample preparation process. The superficial enamel was ground to remove surface irregularities and create a flat enamel surface using a Struers Rotopol 31/Rotoforce 4 polishing unit (Struers Inc., Cleveland, PA, USA). The dentin side of the specimens was ground flat to a uniform thickness with 500-grit silicon carbide grinding paper. The enamel side of the specimens was ground in a series of 1200-, 2400-, and 4000-grit paper. The specimens were then polished using a 1 μm diamond polishing suspension on a polishing cloth. This procedure helped to ensure the removal of surface enamel, which can contain high concentrations of impurities (e.g. F) that can potentially compromise the comparison between the samples. The resulting specimens had a thickness range of 1.9-2.2 mm. Specimens with cracks, hypomineralized (white spot) areas, or other surface flaws were excluded. The prepared specimens were then stored in 100% relative humidity at 4°C until further use.

### Caries lesion creation

*In vitro* incipient caries lesions were created in the specimens by a 48-hour demineralization at 37°C under static conditions and using a solution (40 ml *per* specimen) with the following composition: 0.1 M lactic acid, 4.1 mM CaCl_2_ × 2 H_2_O, 8.0 mM KH_2_PO_4_, and 0.2% *w/v* Carbopol C907 (BF Goodrich Co., Akron, OH, USA), pH adjusted to 5.0 using potassium hydroxide (KOH)^21^. After lesion creation, each specimen was mounted on the end of an acrylic rod (1/4” diameter × 2” long) using cyanoacrylate (Turbo Fuse General Purpose Cyanoacrylate Adhesive, Palm Labs Adhesives, DeBary, FL, USA). All surfaces of the specimen apart from the polished enamel surface were covered with acid-resistant nail varnish.

### Caries lesion Vickers surface microhardness

Four indentations were placed into the formed lesion and by means of a Vickers diamond indenter (2100 HT; Wilson Instruments, Norwood, MA, USA) while using a 200-gram load. Indentations were placed in the center of each specimen, approximately 200 μm apart from one another, with a dwelling time of 15 seconds. The Vickers hardness number (VHN_demin_) of each specimen was calculated using the mean of the length of both diagonals of the four indentations. Only specimens with a VHN_demin_ that was within the range of the mean VHN_demin_ ±2 standard deviation of all specimens were used in this study. Specimens were stratified into treatment groups using VHN_demin_ to ensure no significant differences in the mean VHN_demin_ between groups. Each treatment group contained 18 specimens.

### Study products and groups

Study toothpastes were a SDS-containing toothpaste (Crest Cavity Protection; Procter & Gamble, Mason, OH, USA; 1150 ppm fluoride as sodium fluoride, surfactant: sodium dodecyl sulfate, abrasive: hydrated silica) and CAPB-containing toothpaste (Sensodyne ProNamel; GlaxoSmithKline, Parsippany, NJ, USA; 1150 ppm fluoride as sodium fluoride, surfactant: cocamidopropyl betaine; abrasive: hydrated silica). The studied CHX mouthwash was Paroex (GUM, Schaumburg, IL, USA; 0.12% chlorhexidine gluconate).

In a total of ten study groups, specimens of five groups were treated with the SDS-containing toothpaste and the other five with the CAPB-containing toothpaste. CHX rinsing was performed immediately after toothpaste treatment, or 15, 30 or 60 minutes thereafter. Two negative control groups, which received toothpaste treatments but not CHX or any other rinse, were also included.

### pH-cycling

**Figure 1:**
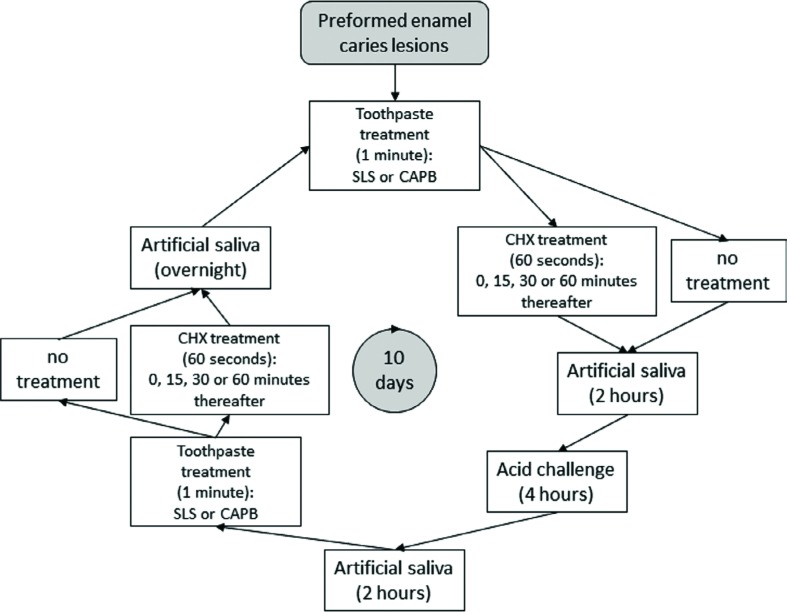
Schematic representation of the pH-cycling model

This study employed an established pH-cycling model based on that by White^39^ (1987) with a pH-cycling phase duration of 10 d. The daily pH-cycling schedule can be found in Figure 1. Toothpaste slurries were prepared using artificial saliva at a 1:2 dilution ratio. The artificial saliva had the following composition: 2.20 g/l gastric mucin, 1.45 mM CaCl_2_ × 2 H_2_O, 5.42 mM KH_2_PO_4_, 6.50 mM NaCl and 14.94 mM KCl, pH adjusted to 7.0 using KOH.

The specimens were placed for 60 seconds into a CHX solution 15, 30, 60 minutes or immediately after each fluoride treatment, or not at all (negative control). During the waiting periods, specimens were placed into artificial saliva. Specimens were briefly rinsed under running deionized water (approximately 2-3 seconds) whenever they were being transferred from one solution to another. This study was conducted at room temperature. After the last treatment with 10 days of pH-cycling, the specimens were placed into artificial saliva for 30 minutes before being rinsed with deionized water.

### Post pH-cycling surface microhardness

After completion of the pH-cycling phase, a second set of four indentations were placed exactly as described above, although approximately 200 µm to the right of the lesion baseline indentations, yielding VHN_remin_. The change in VHN, ∆VHN, was calculated as follows: $∆\text{VHN}={\text{VHN}}_{\text{remin}}–{\text{VHN}}_{\text{demin}}$ (∆VHN>0 is indicative of remineralization; ∆VHN<0 is indicative of further demineralization)^32^.

### Statistical considerations

#### Sample size calculation

Based on prior data, the coefficient of variation estimated was 0.7. With a sample size of 18 *per* toothpaste-timing of CHX rinse combination, the study was designed to have 80% power to detect a 1.85× difference between any two groups, assuming two-sided tests each conducted at a 5% significance level.

### Statistical analysis

The effects of toothpaste and timing of CHX rinse on ∆VHN for remineralization of early caries lesions were analyzed using two-way ANOVA, which included fixed factors for toothpaste, timing of CHX rinse, and their interaction. Pair-wise comparisons between treatments were made using Fisher’s Protected Least Significant Differences. A 5% significance level was used for all tests. The distribution of the measurements were examined, and no transformation of the data was necessary.

## Results

A total of 200 specimens were initially demineralized, of which 180 specimens were selected for this study as described above. VHN_demin_ and ∆VHN data for each study group as well as the results of the statistical analysis can be found in Table 1. Toothpaste type and treatment waiting time had significant effects on remineralization (p<0.0001 and p=0.0346, respectively). The interaction between toothpaste type and treatment waiting time was not significant (p=0.2031). Lesions treated with the SDS-toothpaste showed less remineralization than those treated with the CAPB-toothpaste (p<0.0001). There was less remineralization for groups with the 0-minute treatment waiting time than for the negative control groups (p=0.0022) and for the 30-minute waiting time (p=0.0233) as well as a similar trend for the 60-minute waiting time (p=0.07).

**Table 1:** Microhardness data (mean±standard deviation) and results of the statistical analysis

Toothpaste	Waiting Time	VHNdemin	∆VHN	Statistical Comparisons
SDS	0 minutes	49.9±16.3	9.0±15.2	D*
	15 minutes	49.6±15.2	14.8±18.7	CD
	30 minutes	49.4±14.1	27.6±23.9	C
	60 minutes	49.4±13.2	13.8±12.5	CD
	Control	49.4±13.2	19.1±15.0	C
CAPB	0 minutes	49.4±13.1	53.1±34.5	B
	15 minutes	49.3±13.1	65.2±37.1	AB
	30 minutes	49.4±13.0	62.6±28.7	A
	60 minutes	49.4±13.0	70.5±35.7	AB
	Control	49.4±13.5	81.5±23.9	A

* Different letters, in descending order from A (most remineralization) to D (least remineralization), indicate significant differences in ∆VHN values between study groups.

Pair-wise comparisons between treatments were made using Fisher's Protected Least Significant Differences.

## Discussion

This *in vitro* study concerned with determining interactions between sodium fluoride, toothpaste surfactants and CHX on enamel caries lesion remineralization, with waiting time (i.e. time between fluoride toothpaste and CHX applications) being added as an additional variable. The chosen chemical model did not include a microbial aspect, as the attempt was to mimic remineralization of smooth surface caries lesions, which, if kept clean, do not present biofilm accumulation.

This study indicated that treatment with the CAPB-toothpaste resulted in significantly more remineralization than with the SDS-toothpaste, therefore rejecting the null hypothesis b). Ambarkova, et al.^2^ (2011) indicated more remineralization after treatment with a most likely identical CAPB-toothpaste to the one used presently compared with other fluoride toothpastes, although their study used a toothpaste with a higher fluoride concentration (1450 ppm). The hydroxyapatite in enamel consists mainly of calcium, phosphate and hydroxyl groups. On the surface of hydroxyapatite crystals, however, more phosphate groups are exposed than calcium atoms. Therefore, enamel has a negative surface charge^10^. SDS is an anionic molecule^8^, while CAPB is considered a zwitterion, meaning it contains both positive and negative electrical charges. Although there are no prior comparative caries studies between toothpastes with different surfactants, this may indicate that CAPB-toothpastes have less electrostatic attraction to calcium binding sites at the enamel surface than SDS-toothpastes, which increases the number of binding sites for fluoride and may therefore explain their greater remineralizing potential. Furthermore, surface-bound surfactants affect ion transport in and out of the lesion. The greater affinity of SDS to the lesion surface compared with CAPB may therefore also hamper ion transport into the lesion and provide another explanation for these observations. Furthermore, surfactants, in particular SDS, may also block active sites of crystal growth by acting as a crystal poison due to their high affinity to calcium.

CHX is a large dicationic molecule and can therefore adsorb onto negatively charged surfaces^19^, such as enamel. However, CHX also has a great affinity for the negatively charged SDS, leading to its desorption from the enamel surface and “inactivation” of CHX, and therefore, reduces its availability on the tooth surface. For both CAPB- and SDS-toothpastes, there was less remineralization after pH-cycling with immediate CHX treatment than for the negative control group, in which no CHX treatment was used. Furthermore, there was significantly less remineralization with immediate CHX treatment than with the 30 minutes waiting time. This suggests CHX interferes with remineralization and, potentially, with fluoride mode of action. However, this effect can be mitigated by extending the waiting time between fluoride and CHX treatments. The nature of this interaction is presently unknown and warrants further research. It is likely that CHX slowly desorbs loosely bound fluoride from the enamel surface and thereby minimizes remineralization. Increasing the waiting time would allow more fluoride to be incorporated into the enamel structure and thereby preempt the effect of CHX. Likewise, rinsing with water alone immediately after a fluoride treatment has the potential to remove loosely bound fluoride and lessen its anticaries effects^11^ ^,^ ^12^ ^,^ ^23^ ^,^ ^28^. These aspects can potentially explain our findings.

In the past, research on CHX was more concerned with demonstrating antimicrobial and direct lesion effects rather than focusing solely on the latter. Timmons, et al.^34^ (2007) fixed artificial caries lesions on crowns that were placed on prepared patient teeth. Patients were then instructed to brush using a placebo toothpaste, a fluoride toothpaste, or a fluoride toothpaste followed by CHX. Their study showed CHX used in combination with fluoride toothpaste was no more effective in reducing dental caries than fluoride toothpaste alone. Altenburger, et al.^1^ (2006) evaluated the ability of CHX/NaF and CHX rinses to remineralize demineralized enamel specimens *in situ*. No differences were observed; however, their study did not investigate the effect of CHX on fluoride’s ability to remineralize lesions, as there was no fluoride only group. An *in vivo* study on a twice-daily CHX and once-daily fluoride rinse regimen by Ullsfoss, et al.^36^ (1994) utilized plaque-retaining bands on premolars planned for extraction. The authors were able to demonstrate additive effects for the combined CHX and fluoride regimen; however, their model was concerned with prevention of demineralization rather than the enhancement of remineralization. While these studies do not provide a rationale for our findings, they show CHX is not only being used for the prevention of periodontal disease but also in caries prevention. This study, however, has shown that care must be taken when applying CHX in relation to fluoride and that the type of toothpaste needs to be chosen carefully to maximize the anticaries benefits of fluoride.

The following study limitations must be borne in mind when interpreting these data. Hardness techniques cannot directly determine the extent of mineral loss or gain; however, the measured increases in surface hardness are due to remineralization. Furthermore, this study was conducted *in vitro* and did not consider the oral soft tissues which serve as reservoirs for fluoride. In an *in vivo* environment, fluoride and CHX may be retained on the tongue, and because of its large surface area, this may increase the availability of the active agents and impact remineralization in a different way than observed presently. Additionally, the chosen chemical model did not allow to determine potential interactions between fluoride, surfactants and CHX on cariogenic biofilms. Lastly, caries lesions vary considerably in their mineral distributions and severity and do not necessarily present a surface layer^3^. Our findings cannot be generalized until studies on a wide range of enamel caries lesions is conducted. Therefore, future research, and in particular *in situ*- and *in vivo*-type studies would be required to confirm our observations.

## Conclusions

In conclusion, and bearing in mind the limitations of this laboratory study, the absence of CHX as an adjunct to fluoride toothpastes led to greater enamel remineralization, as measured by SMH, compared with immediate use of CHX treatment. This was true for both SDS- and CAPB-toothpastes. Additionally, a 30-minute waiting time for CHX treatment exhibited greater remineralization than immediate CHX treatment. Considering the type of surfactant in the toothpaste, CAPB-toothpastes indicated significantly greater remineralization than SDS-toothpastes at all, CHX treatment waiting times as well as for the negative control. Therefore, CAPB-toothpaste can be recommended to patients at high caries risk or while on CHX treatment of periodontal disease. A 30-minute waiting time for CHX treatment is recommended after brushing.
